# Remodeling of Tight Junctions and Enhancement of Barrier Integrity of the CACO-2 Intestinal Epithelial Cell Layer by Micronutrients

**DOI:** 10.1371/journal.pone.0133926

**Published:** 2015-07-30

**Authors:** Mary Carmen Valenzano, Katherine DiGuilio, Joanna Mercado, Mimi Teter, Julie To, Brendan Ferraro, Brittany Mixson, Isabel Manley, Valerissa Baker, Beverley A. Moore, Joshua Wertheimer, James M. Mullin

**Affiliations:** 1 Lankenau Institute for Medical Research, Wynnewood, PA, 19096, United States of America; 2 Division of Gastroenterology, Lankenau Medical Center, Wynnewood, PA, 19096, United States of America; 3 Department of Biology, Drexel University, Philadelphia, PA, 19104, United States of America; 4 Janssen Research & Development, LLC, Spring House, PA, 19477, United States of America; University of Chicago, UNITED STATES

## Abstract

The micronutrients zinc, quercetin, butyrate, indole and berberine were evaluated for their ability to induce remodeling of epithelial tight junctions (TJs) and enhance barrier integrity in the CACO-2 gastrointestinal epithelial cell culture model. All five of these chemically very diverse micronutrients increased transepithelial electrical resistance (R_t_) significantly, but only berberine also improved barrier integrity to the non-electrolyte D-mannitol. Increases of R_t_ as much as 200% of untreated controls were observed. Each of the five micronutrients also induced unique, signature-like changes in TJ protein composition, suggesting multiple pathways (and TJ arrangements) by which TJ barrier function can be enhanced. Decreases in abundance by as much as 90% were observed for claudin-2, and increases of over 300% could be seen for claudins -5 and -7. The exact effects of the micronutrients on barrier integrity and TJ protein composition were found to be highly dependent on the degree of differentiation of the cell layer at the time it was exposed to the micronutrient. The substratum to which the epithelial layer adheres was also found to regulate the response of the cell layer to the micronutrient. The implications of these findings for therapeutically decreasing morbidity in Inflammatory Bowel Disease are discussed.

## Introduction

There seems little doubt that compromised epithelial barrier integrity plays a role in a wide range of disease in general [1,2] and in Inflammatory Bowel Disease (IBD) in particular [3,4]. The leakage of food and bacterial antigens, bacterial endo- and exotoxins, as well as bacteria themselves from the gastrointestinal lumen into the lamina propria and submucosal spaces, cannot be a benign or even neutral event. If these processes are not causal in IBD, they almost certainly are contributory. A good deal of past and present therapy in IBD has focused on dealing with the inflammatory aftermath of such leakage, attempting to tamp down immunological “brush fires.” A different strategy however may be forthcoming, namely reducing the unregulated paracellular leak that at least contributes to the local proinflammatory state.

Over the past 10 plus years, an increasing number of reports have surfaced in the biomedical literature that certain functional substances exist at very low concentrations in the diet, a subclass of “micronutrients” or “nutraceuticals,” that have the ability to induce structural changes in the tight junctional (TJ) complex that result in a complex that is less leaky [5,6]. This is no mean feat given that an exceedingly wide range of disease processes and pathogens exist capable of inducing changes in the TJ which universally result in *greater* leak [2,7].

Given that a TJ complex can have as many as 28 different integral membrane proteins whose homotypic and heterotypic interactions create the diffusion barrier, the ability of specific micronutrients to engineer a “better,” less-leaky TJ complex is remarkable. The fact that these various micronutrients each achieve this with their own individual, almost signature-like structural/compositional changes to the TJ, is even more impressive. In this study we sought to extend our earlier observations concerning micronutrients and renal epithelial TJ complexes [8], to a gastrointestinal cell line model (CACO-2 BBE) with relevance to IBD, and explore how the state of differentiation of the cell layer affects its TJs’ response to the micronutrients.

We have selected micronutrients that have been reported individually in the literature to display the ability to induce compositional changes in the TJ, and enhance epithelial barrier integrity. They are zinc, indole, butyrate, quercetin, nicotine and berberine, a motley assortment of substances ranging from a trace metal to bacterial metabolites to a bioflavonoid and two alkaloids. Although these micronutrients are each amassing an impressive individual collection of publications reporting TJ “tightening,” they have not-with one exception[8]- been studied collectively in a single model and a single investigation.

The impetus for the study of zinc regarding possible effects on TJ barriers appears to derive from observations concerning the correlation between zinc deficiency and pediatric diarrhea [9]. Zinc deficiency has been observed to compromise barrier integrity of CACO-2 cell layers, a human cell culture model of the small bowel epithelium [10]. Zinc supplementation, on the other hand, improves barrier integrity with very specific effects on TJ complexes [11,12]. Zinc supplementation can also offset the effects of agents such as proinflammatory cytokines that themselves impair barrier integrity [13,14,15]. The short chain fatty acid, butyrate, also improves epithelial barrier integrity, and does so with its own specific modifications of the TJ complex [16,17]. Its clinical history regarding the effectiveness of butyrate enemas for ulcerative colitis treatment made it a compelling choice in the search for substances with beneficial effects on barrier integrity [18]. The heterocyclic aromatic, indole, has been reported to not only increase transepithelial electrical resistance of a gastrointestinal cell line model, but also decrease epithelial production of proinflammatory cytokines that may negatively impact barrier integrity [19]. It is noteworthy that both butyrate and indole are bacterial metabolites in the GI lumen, from fiber and tryptophan respectively, and thus also point to the rising interest in probiotics as a source of GI barrier enhancement [20,21]. Nicotine has been shown to improve CACO-2 barrier integrity regarding electrical resistance and fluorescein permeability [22]. Another plant-derived alkaloid, berberine, has likewise been reported to reduce TJ permeability in the CACO-2 cell layer [23,24]. The bioflavonoid, quercetin, a component of green and black teas, also has properties of epithelial barrier improvement at micromolar concentrations, as do certain other compounds of the bioflavonoid class [25]. As is true of the other dietary components listed above, quercetin enhancement of epithelial barrier integrity appears to occur with structural/compositional modifications of the TJ complex [25,26,27].

## Material and Methods

### Cell Culture

The CACO-2 BBE cell culture, an epithelial cell line derived from human colon adenocarcinoma [28,29], was obtained from ATCC, and was used between passages 52 and 68. Upon confluence, cells were passaged on a weekly basis by trypsinizination (0.25% trypsin and 2.2 mM EDTA [Corning Cellgro]) and were seeded at 5 x 10^5^ cells/Falcon 75-cm^2^ culture flask with 25 ml of Dulbecco-s Modified MEM (Minimum Essential Medium) (Corning Cellgro) supplemented with 2mM L-Glutamine (Corning Cellgro), 1% Non Essential Amino Acids (Corning Cellgro), 1mM Sodium Pyruvate (Corning Cellgro) and 10% defined fetal bovine serum (HyClone). Cultures were incubated at 37°C in 95% air-5% CO_2_ atmosphere.

### Media Supplementation with Nutraceuticals

For each nutraceutical, with the exception of quercetin, a stock solution was prepared. A serial dilution was then done in culture medium to attain desired working concentrations for treatment of cell layers. Prior to supplementation, the media was filter sterilized with a 0.2 μm sterile syringe (Corning). For zinc, a stock solution (100 mM) was made from zinc sulfate heptahydrate (Fisher Chemical) in deionized distilled water. A butyrate stock solution (400 mM) was made from sodium butyrate (Sigma-Aldrich) in deionized distilled water. A berberine (Sigma-Aldrich) stock solution (2.7 mM) was also prepared in deionized distilled water, but was made each day at the time of use. In the case of indole (Sigma-Aldrich), a 400 mM stock solution was prepared in absolute ethanol. With quercetin (Sigma-Aldrich), dry chemical was added directly to complete culture medium to make up a working concentration (400 μM) that was applied directly to cells. Solubilization of quercetin in medium at 400 μM required warming medium to 38°C for 40 minutes with constant stirring. Lower concentrations were prepared simply by serial dilution in complete medium. Proper solvent controls were performed in all experiments.

### Transepithelial Electrophysiology and Permeability

Cells were seeded into sterile Millipore Millicell polycarbonate (PCF) permeable supports (30 mm diameter with 0.4 μm pore size) on day 0 at a seeding density of 5 x 10^5^ cells/insert. Three or four sterile Millicell PCF inserts were placed into a 100 mm petri dish. On day 1, all cell layers were refed (2 ml apical/15 ml basal-lateral) with control medium containing 50 U/ml penicillin and 50 μgms/ml streptomycin, followed by refeedings every 2–3 days until treatment. Depending upon the specific nutraceutical, cells were fed on both cell surfaces with medium supplemented with the appropriate micronutrient for either 12–17, 24 or 48 hour treatment (depending upon agent and based upon published reports), followed by electrophysiological measurements and radiotracer flux studies with ^14^C-D-mannitol.

On the day of transepithelial experiments, the cell layers were re-fed with fresh control medium and allowed to incubate at 37°C for 1 to 1.5 hours prior to electrophysiological readings. Transepithelial potential difference (PD), transepithelial electrical resistance (R_t_), and short-circuit current (I_sc_) were measured using 1 sec, 40 μamp direct current pulses, and calculated using Ohm’s law. As soon as electrical measurements were completed, the basal-lateral medium was aspirated and replaced with 15 ml of medium containing 0.1 mM, 0.20 μCi/ml ^14^C-D-mannitol (Perkin-Elmer, Boston, MA) and incubated at 37°C. Triplicate basal-lateral medium samples (50 μl) were taken for liquid scintillation counting (LSC) for specific activity determination. Duplicate samples (100 μl) were taken from the apical side at 45, 90, and 135 min for LSC to determine flux rates. The media lost due to sampling from the apical compartment was replaced with fresh medium of the same sample volume. The flux rate (Jm) (in cpm/min/cm^2^ and picomoles/min/cm^2^) was calculated for the ^14^C-D-mannitol diffusing across the cell layer.

### Analyses of Tight Junctional Proteins

Cells in 75 cm^2^ culture flasks were allowed to grow to confluence, and then were re-fed with media containing the various nutraceuticals under study at the concentrations that provided maximal barrier enhancement and for the same time periods used in the permeability studies. After the specified incubations, cell layers were washed 2X in 4°C phosphate-buffered saline (PBS) and then harvested by physical scraping into lysis buffer, followed by sonication and ultra centrifugation. Samples of these fractions were analyzed by PAGE using a 4–20% gradient Novex Tris-glycine gel at 125V for 1 hr 45 min. (8% Tris-glycine gels were used in the cases of occludin and tricellulin). Precision Plus Kaleidoscope Protein Standards (Biorad, Inc.) were also included in each gel. Proteins were transferred at 30 V for 2 hr from the gel to a PVDF membrane. The membranes were then washed three times with PBS-T (0.3% Tween-20) for 10 min each and blocked with 5% milk/PBS-T for 1 hr at RT. Membranes were incubated with the specific primary antibody (anti-claudin-1, -2, -4, -5, anti-occludin, anti-tricellulin [Life Technologies]) at 1.0 μg /ml in 5% milk/PBS-T overnight at 4°C then 2 hr at RT. (For occludin, tricellulin and claudins -3 and -7, there was only a 2 minute incubation with the primary antibody at room temperature). The membranes were washed with PBS-T 3X for 10 minutes each, then incubated with secondary antibody (rabbit anti-mouse or goat anti-rabbit IgG labeled with horseradish peroxidase (Southern Biotechnology) for 1 hr at RT. Membranes were washed with PBS-T (4X for 10 minutes each), then treated for 1 min with Western Lighting-ECL chemiluminescence reagents (Perkin Elmer). The membranes were then exposed to HyBlot CL autoradiography film (Denville Scientific) which was developed in a Kodak M35A X-OMAT processor. Band densities were quantified by densitometry. Band densities of nutraceutical-treated cell samples were compared against normalized averages of corresponding control band densities.

### Analysis of Claudin Transcript Expression by RT-PCR

CACO-2 cells in 75 cm^2^ culture flasks were grown to confluence and then re-fed with media containing 5.0 mM butyrate. Cells were cultured for 4, 12, 24, 48, or 72 hours, at which time the media was removed, cells were washed, and then lysed in RLT/ βME buffer, and flash-frozen at -80^oC^. Cell lysates were thawed at 37°C and total RNA was isolated using the RNeasy Mini Kit (Qiagen). RNA concentration and purity were assessed by Nanodrop ND-8000. cDNA was prepared with qScript cDNA SuperMix (Quanta Biosciences) using 400 ng total RNA in a 20 uL reaction. After cDNA synthesis, 18 ng of cDNA was analyzed using Taqman Universal PCR Master Mix (Applied Biosystems) and the following primers from Applied Biosystems: GAPDH (4332649), Claudin-2 (Hs00252666_s1), and Claudin-7 (Hs00600772_m1). Fold-change in gene expression was calculated for butyrate-treated samples compared to appropriate vehicle control samples at the same time-point.

### Statistics

For electrophysiology, radiotracer flux and protein chemistry studies, nutraceutical-treated cell samples were compared against appropriate matched controls within the same experiment. All data is expressed as the mean ± standard error of the mean (SEM) with the number of replicates provided for each set of studies. Differences between means are evaluated by Student’s t tests for two groups, or by one-way analysis of variance (ANOVA) followed by Dunnett’s post-hoc testing for multiple comparisons.

## Results

### Effects of the Various Micronutrients on Epithelial Barrier Integrity

**Fig 1:**
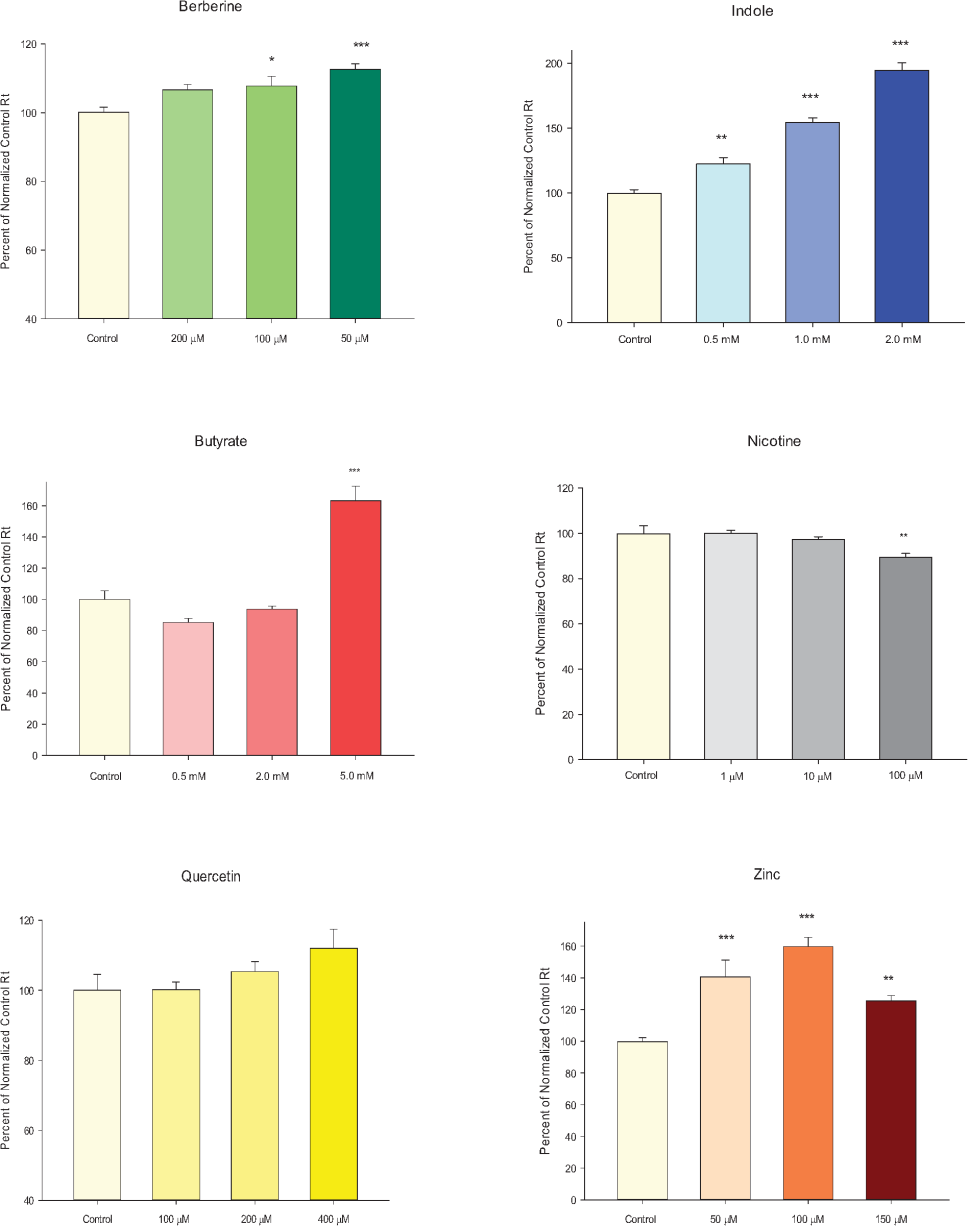
The effect of six micronutrients on CACO-2 transepithelial electrical resistance. 1A: 7-Day post-confluent CACO-2 cell layers on Millipore PCF filters were refed in control medium (apical and basal-lateral compartments) or medium containing 50μM, 100μM, or 200μM berberine chloride 12–17 hrs prior to electrical measurements. Data shown represents the mean ± standard error of 9 cell layers per condition (3 experiments, 3 cell layers per experiment). 1B: 7-Day post-confluent CACO-2 cell layers on Millipore PCF filters were refed, as above, in control medium or medium containing 1μM, 10μM, or 100μM Nicotine 48 hrs prior to electrical measurements. Data shown represents the mean ± standard error of 6 cell layers per condition (2 experiments, 3 cell layers per experiment). 1C: 4-Day post-confluent CACO-2 cell layers on Millipore PCF filters were refed, as in A, in control medium or medium containing 0.5mM, 2.0mM, or 5mM sodium butyrate 72 hrs prior to electrical measurements. Data shown represents the mean ± standard error of 6 cell layers per condition (2 experiments, 3 cell layers per experiment). 1D: 7-Day post-confluent CACO-2 cell layers on Millipore PCF filters were refed, as in A, in control medium or medium containing 100μM, 200μM, or 400μM quercetin 48hrs prior to electrical measurements. Data shown represents the mean ± standard error of 6 cell layers per condition (2 experiments, 3 cell layers per experiment). 1E: 7-Day post-confluent CACO-2 cell layers on Millipore PCF filters were refed, as in A, in control medium or medium containing 0.5mM, 1.0mM, or 2.0mM indole 48hrs prior to electrical measurements. Data shown represents the mean ± standard error of 9 cell layers per condition (3 experiments, 3 cell layers per experiment). 1F: 7-Day post-confluent CACO-2 cell layers on Millipore PCF filters were refed, as in A, in control medium or medium containing 50μM, 100μM, or 150μM zinc sulfate 48hrs prior to electrical measurements. Data shown represents the mean ± standard error of 12 cell layers for both the control and 100μM conditions, and 4 cell layers for both the 50μM and 150μM conditions. In all cases, data represents the percent of control resistance normalized for each experiment. * indicates P < 0.05; ** indicates P < 0.01; *** indicates P < 0.001 (one-way ANOVA followed by Dunnett’s post hoc testing versus control).

**Fig 2:**
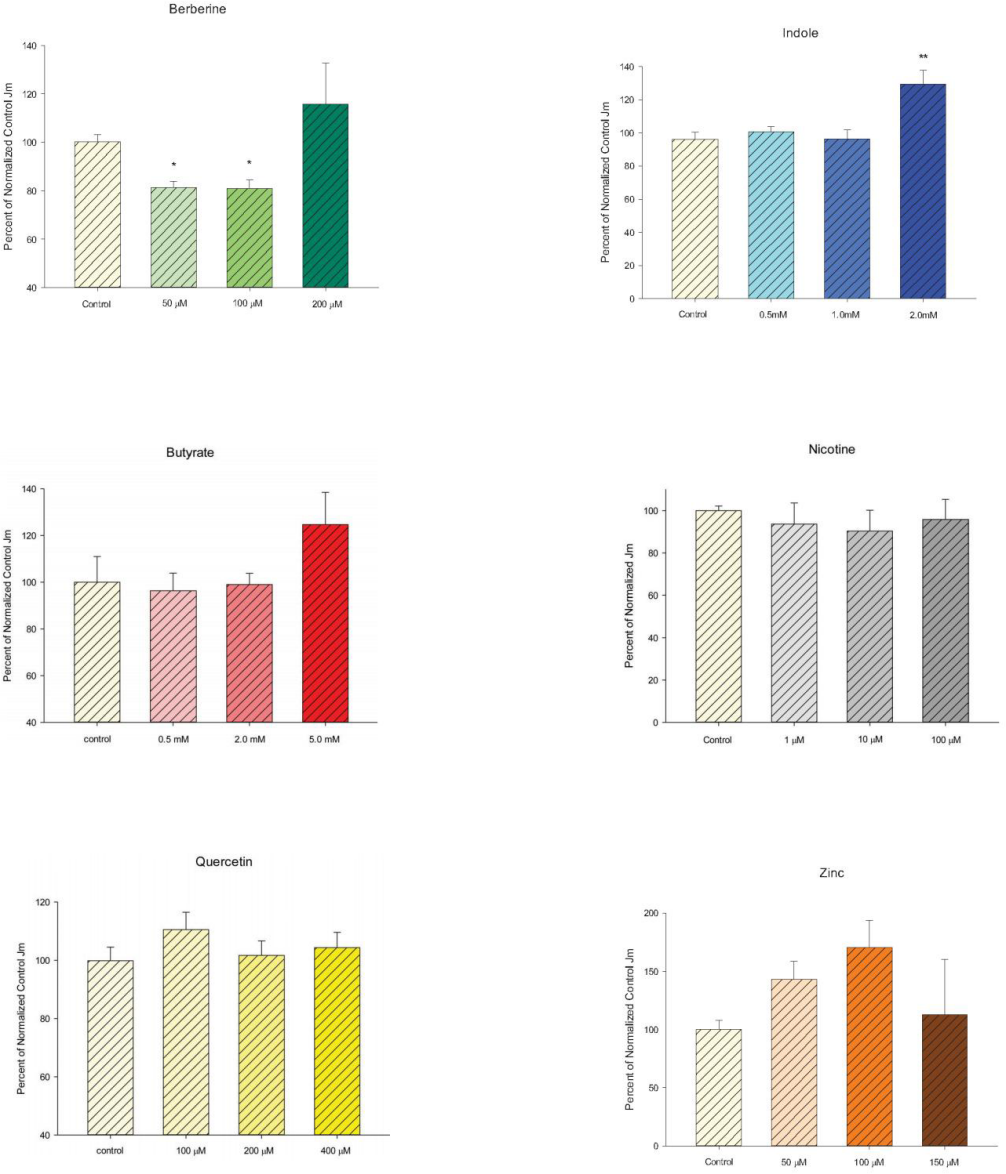
The effect of six micronutrients on CACO-2 transepithelial flux of ^14^C-D-Mannitol. In all cases, after electrical measurements, the same CACO-2 cell layers represented in Fig 1 were used to perform radiotracer flux studies with 0.1mM, 0.20 μCi/ml ^14^C-D-mannitol, as described in Materials and Methods. Data represents the percent of control flux rate normalized for each experiment, and is expressed as the mean ± standard error for the total number of cell layers per condition, as detailed in Fig 1. * indicates P < 0.05, ** indicates P < 0.01 and *** indicates P < 0.001 (one-way ANOVA followed by Dunnett’s post hoc testing versus control).

Following the work of other research groups, we had previously shown that a chemically, highly diverse group of micronutrients could exert similar action in enhancing the barrier integrity of an epithelial cell layer [8]. The effect could be to elevate R_t_, to decrease J_m_ or both. Where we had previously shown this to be true for a renal epithelial cell layer, we here show it to be true for the human gastrointestinal (CACO-2) cell layer. Zinc, quercetin, berberine, indole and butyrate all significantly elevated CACO-2 R_t_ for at least one of the concentrations tested (Fig 1), under conditions described in the figure legend. (The chosen conditions, regarding both micronutrient concentration as well as duration of exposure to micronutrient, tracked reports on the use of these micronutrients in the published literature). Only nicotine failed to affect barrier integrity at any of the concentrations used. In the cases of butyrate and indole, elevations of R_t_ as much as 100% could be observed. Only berberine, however, was able to simultaneously both increase R_t_ and decrease J_m_ significantly, thus evidencing improvement of barrier integrity to two very different solute classes (Fig 2). Considering that these changes in barrier integrity represent changes from the basal state, not changes from a compromised state, the increases in R_t_ and/or decreases in J_m_ are quantitatively quite noteworthy.

### Effects of the Various Micronutrients on Tight Junctional Proteins

The 5 micronutrients that altered barrier integrity significantly were then studied for the corresponding changes that they induced in the abundance of various integral TJ proteins in whole cell lysates. The concentrations of micronutrients chosen were those that displayed the greatest elevation of R_t_ with minimal or no increase in J_m_. The durations of exposure were those used in the barrier integrity studies of Figs 1 and 2. As shown in Table 1, each of the 5 micronutrients exhibited a unique, signature-like effect concerning alterations of TJ protein abundance. Significant increases of TJ protein abundance are highlighted in green while decreases are highlighted in yellow to accentuate the distinct patterns associated with each micrnutrient. In certain cases, changes were highly substantial, as in the over 80% decreases of claudin-2 caused by berberine and butyrate, and over 300% increases of claudins -5 and -7 caused by quercetin and butyrate, respectively. The highly unique patterns of induced changes suggest distinct intracellular signaling pathways at work in the case of each micronutrient.

**Table 1:** Effects of Individual Micronutrients on CACO-2 Tight Junctional Proteins.

	% of matched control cell layers	% increase/decrease
**ZINC**		
Claudin 1:	100% ± 10% (NS)	0%
Claudin 2:	86% ± 15% (NS)	14% decrease
Claudin 3:	81% ± 4% (P < 0.08)	19% decrease
Claudin 4:	106% ± 12% (NS)	6% increase
Claudin 5:	106% ± 9% (NS)	6% increase
Claudin 7:	74% ± 11% (NS) P = 0.085	26% decrease
Occludin:	96% ± 13% (NS)	4% decrease
Tricellulin:	88% ± 7% (NS)	12% decrease
**Quercetin**		
Claudin 1:	69% ± 9% (P < 0.06)	31% decrease
Claudin 2:	264% ± 38% (P < .006)	164% increase*
Claudin 3:	110% ± 14% (NS)	10% increase
Claudin 4:	253% ± 12% (P < .001)	153% increase*
Claudin 5:	457% ± 28% (P < .001)	357% increase*
Claudin 7:	72% ± 14% (NS) P = 0.091	28% decrease
Occludin:	112% ± 9% (NS)	12% increase
Tricellulin:	91% ± 1% (P < .008)	9% decrease*
**Butyrate**		
Claudin 1:	61% ± 16% (P < 0.1)	39% decrease*
Claudin 2:	10% ± 4% (P < .001)	90% decrease*
Claudin 3:	116% ± 14% (NS)	16% increase
Claudin 4:	130% ± 22% (NS)	30% increase
Claudin 5:	110% ± 15% (NS)	10% increase
Claudin 7:	476% ± 106% (P < .01)	376% increase*
Occludin:	215% ± 44% (P < 0.06)	115% increase
Tricellulin:	124% ± 9% (NS)	24% increase
**BERBERINE**		
Claudin 1:	114% ± 9% (NS)	14% increase
Claudin 2:	20% ± 5% (P < .001)	80% decrease*
Claudin 3:	144% ± 18% (P < 0.10)	44% increase
Claudin 4:	118% ± 12% (NS)	18% increase
Claudin 5:	143% ± 10% (P < 0.06)	43% increase
Claudin 7:	104% ± 25% (NS)	4% increase
Occludin:	143% ± 32% (NS)	43% increase
Tricellulin:	124% ± 10% (P < 0.08)	24% increase
**INDOLE**		
Claudin 1:	92% ± 8% (NS)	8% decrease
Claudin 2:	72% ± 8% (P < 0.06)	28% decrease
Claudin 3:	110% ± 6% (NS)	10% increase
Claudin 4:	120% ± 11% (NS) P = 0.074	20% increase
Claudin 5:	162% ± 13% (P < 0.04)	62% increase*
Claudin 7:	95% ± 5% (NS)	5% decrease
Occludin:	126% ± 18% (NS)	26% increase
Tricellulin:	130% ± 8% (P < 0.08)	30% increase

Summary of effects of various micronutrients on a panel of eight tight junctional proteins in CACO-2 cell layers. Post-confluent CACO-2 cell layers in Falcon 75 cm^2^ culture flasks were refed on both sides with control medium or medium containing 100μM Zinc 48 hrs before harvesting in lysis buffer. These total cell lysates were analyzed by PAGE followed by immunoblotting as described in Methods. Immunoblots were probed with primary antisera against specific tight junctional antigens also as described. Densitometry was performed on developed blots to quantitate band densities. Data shown represents the mean ± standard error for an n = 3 cell layers in all cases. P values are listed for instances of statistical significance (Student’s t test [two tailed] to at least the P<0.05 level). NS indicates non significance. This same procedure was used for quercetin (400μM), butyrate (5.0mM), berberine (100μM), and indole (1.0mM) at the concentrations that provided maximal barrier enhancement and for the same time periods used in Figs 1 and 2. Significant increases are highlighted in green while significant decreases are highlighted in yellow to emphasize the distinct overall pattern specific to each micronutrient.

### The Influence of the Differentiation State of the CACO-2 Cell Layer on Micronutrient Effects

**Fig 3:**
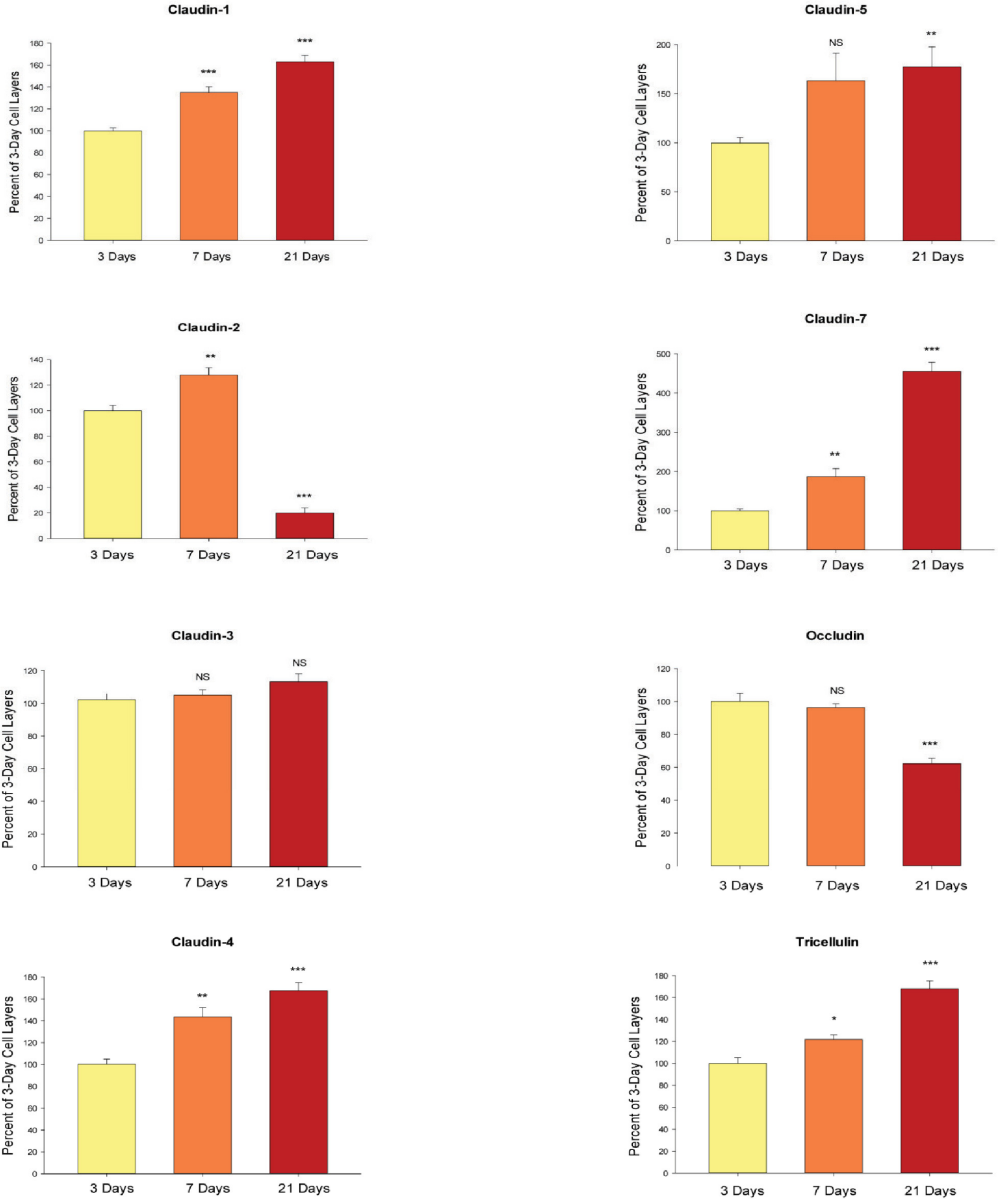
Change of tight junctional proteins as a function of state of differentiation of the CACO-2 cell layer. The relative changes in the abundance of eight tight junctional proteins occurring as a function of days post confluence of the cell layer. 3-day, 7-day, and 21-day post-confluent CACO-2 cell layers in Falcon 75 cm^2^ culture flasks, having been refed with control medium at confluence and every 2–3 days thereafter with control medium, were harvested in lysis buffer. Further steps were performed as described in Table 1. Data represents the percent of 3-day cell layers, and is expressed as the mean ± standard error for an n = 3 cell layers in all cases. NS indicates non significance. * indicates P < 0.05; ** indicates P < 0.01; *** indicates P < 0.001 (one-way ANOVA followed by Dunnett’s post hoc testing versus day 3).

We observed that the state of differentiation of the cell layer is an important determinant of the response of the cell layer’s barrier integrity to micronutrients. This has not only biomedical significance but also procedural importance concerning the exact manner in which one performs these studies. It is well known that the CACO-2 cell layer slowly differentiates over time once the culture achieves confluence, and that as many as 21 days (post-confluence) can be needed for certain differentiated properties to appear [30]. In Fig 3, we show that TJ proteins are no different in this respect, with changes in the relative abundance of these proteins occurring as a function of days post-confluence. For cells seeded onto Millicell PCF membranes, this equates to days post-seeding, as cells are seeded into PCF units at a confluent cell density. For most of the TJ proteins, a graded increase in abundance was observed (claudins -1, -4, -5, -7 and tricellulin), but with certain noteworthy exceptions. Claudin-2 abundance dramatically plummets by day 21. On the other hand, claudin-3 levels showed absolutely no change as a function of days post-confluence of the cell layer. Interestingly, a significant decrease in occludin levels was also observed by 21 days. In summary, the TJ complex appears to be changing as the cell layer differentiates. This is seen in a functional manner in Table 2, where by 7 days, a maximal elevation of R_t_ and decrease in mannitol permeability (J_m_) is seen. Further improvement in these two parameters of barrier integrity is not evident after day 7, although, as seen in Fig 3, dramatic changes in overall abundance of TJ proteins are still ongoing. Differentiation of the cell layer is still continuing after day 7, as shown by the graded significant increases in I_sc_ from 3 to 7 to 21 days (Table 2).

**Table 2:** Effect of Days Post-Seeding on Transepithelial Parameters of CACO-2 Cell Layers.

	3 Day	7 Day	21 Day
Transepithelial Resistance	369 + 72	525 + 13 *	491 + 9
Short Circuit Current	1.0 + 0.2	1.9 + 0.1**	2.6 + 0.2***
Mannitol Flux Rate	0.73 + 0.15	0.27 + 0.03*	0.23 + 0.03**

3-day, 7-day, and 21-day CACO-2 cell layers were refed in control medium the day after they were seeded onto Millicell PCFs, as well as, every two to three days until electrophysiology measurements and ^14^C-D-mannitol flux studies were completed as described in Materials and Methods. Data shown for transepithelial resistance and short circuit current expressed as the mean ± standard error of 8 cell layers per condition (2 experiments, 4 cell layers per condition). Data shown for mannitol flux rate is expressed as the mean ± standard error of 4 cell layers per condition (1 experiment, 4 cell layers per condition).

* indicates P<0.05 (n = 4) vs. 3-day

** indicates P<0.01 (n = 8) vs. 3-day

*** indicates P < 0.001 vs 3-day (one-way ANOVA followed by Dunnett’s post hoc testing versus day 3 as control).

Note that SEM are 20% of means for all three parameters for 3-day cell layers but fall on average to only 7% for 7-day and 21-day cell layers, reflecting the relative cell layer heterogeneity inherent in 3-day cultures.

**Fig 4:**
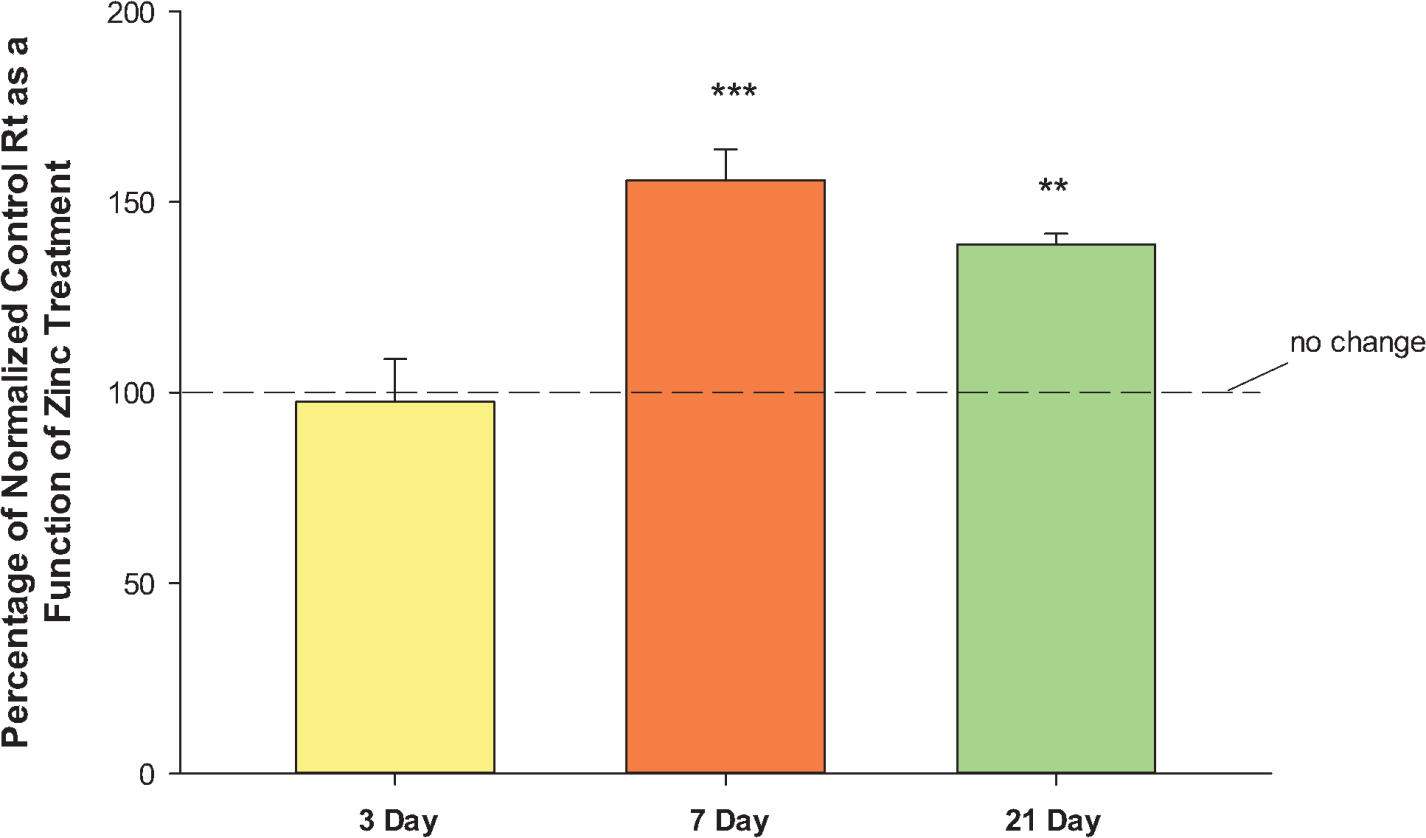
Effect of zinc on CACO-2 transepithelial electrical resistance as a function of the differentiation state of the CACO-2 cell layer. 3-day, 7-day, and 21-day CACO-2 cell layers were treated on the apical and basal-lateral sides with 100μM zinc for 48 hrs before electrical measurements. Data represents the percentage of normalized control resistance (2 experiments, 4 cell layers per condition per experiment) as a function of zinc treatment. Data shown is expressed as the mean ± standard error of 8 cell layers per condition. ** indicates P < 0.01 (21-day vs. 3-day); *** indicates P < 0.001 (one-way ANOVA followed by Dunnett’s post hoc testing versus day 3).

**Fig 5:**
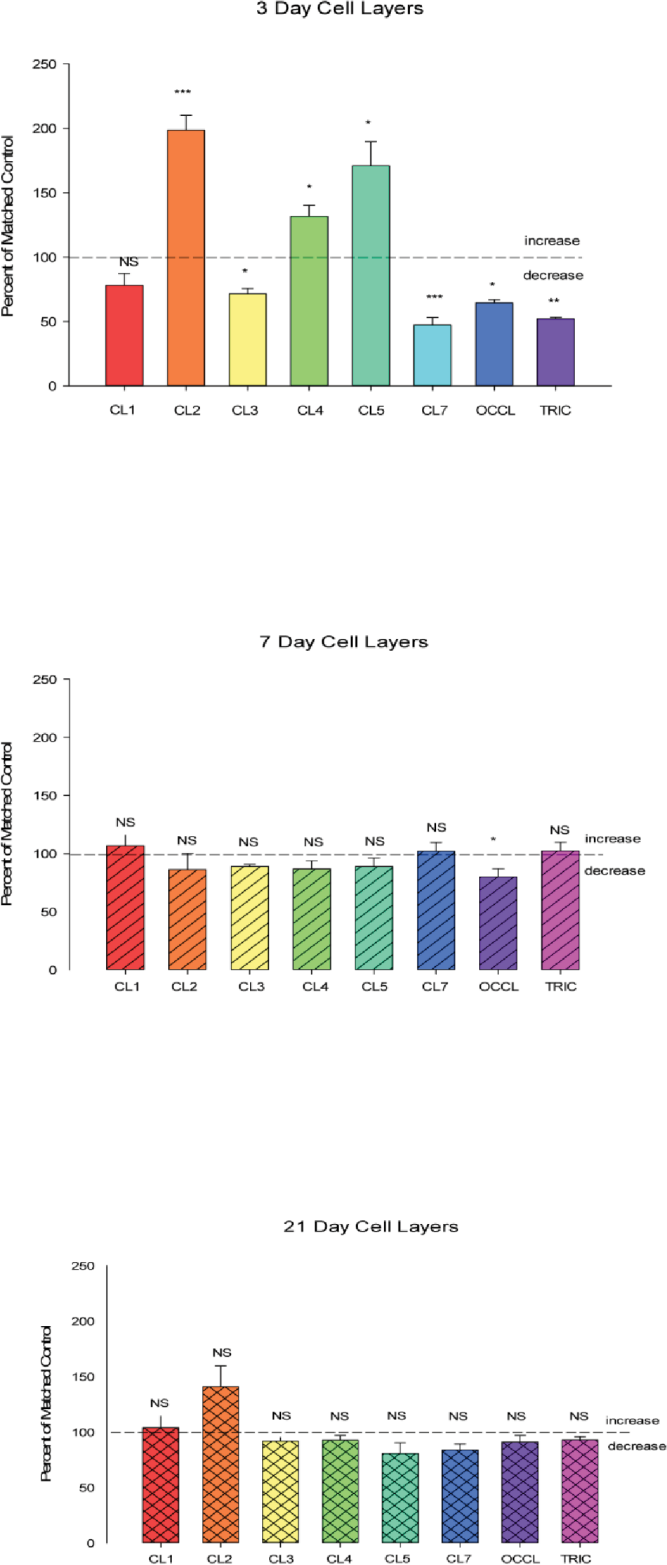
Effect of zinc on CACO-2 tight junctional proteins as a function of the differentiation state of the CACO-2 cell layer. 3-day, 7-day, and 21-day post-confluent CACO-2 cell layers in Falcon 75 cm^2^ culture flasks were refed with control medium or medium containing 100μM Zinc 48 hrs before harvesting in lysis buffer. Further steps were performed as described in Table 1. Data represents the percent of the zinc condition relative to the normalized control for each age cell layer. Data shown is expressed as the mean ± standard error for an n = 4 cell layers in all cases. NS indicates non significance. * indicates P < 0.05; ** indicates P < 0.01; *** indicates P < 0.001 (Student’s t test, two-tailed).

**Fig 6:**
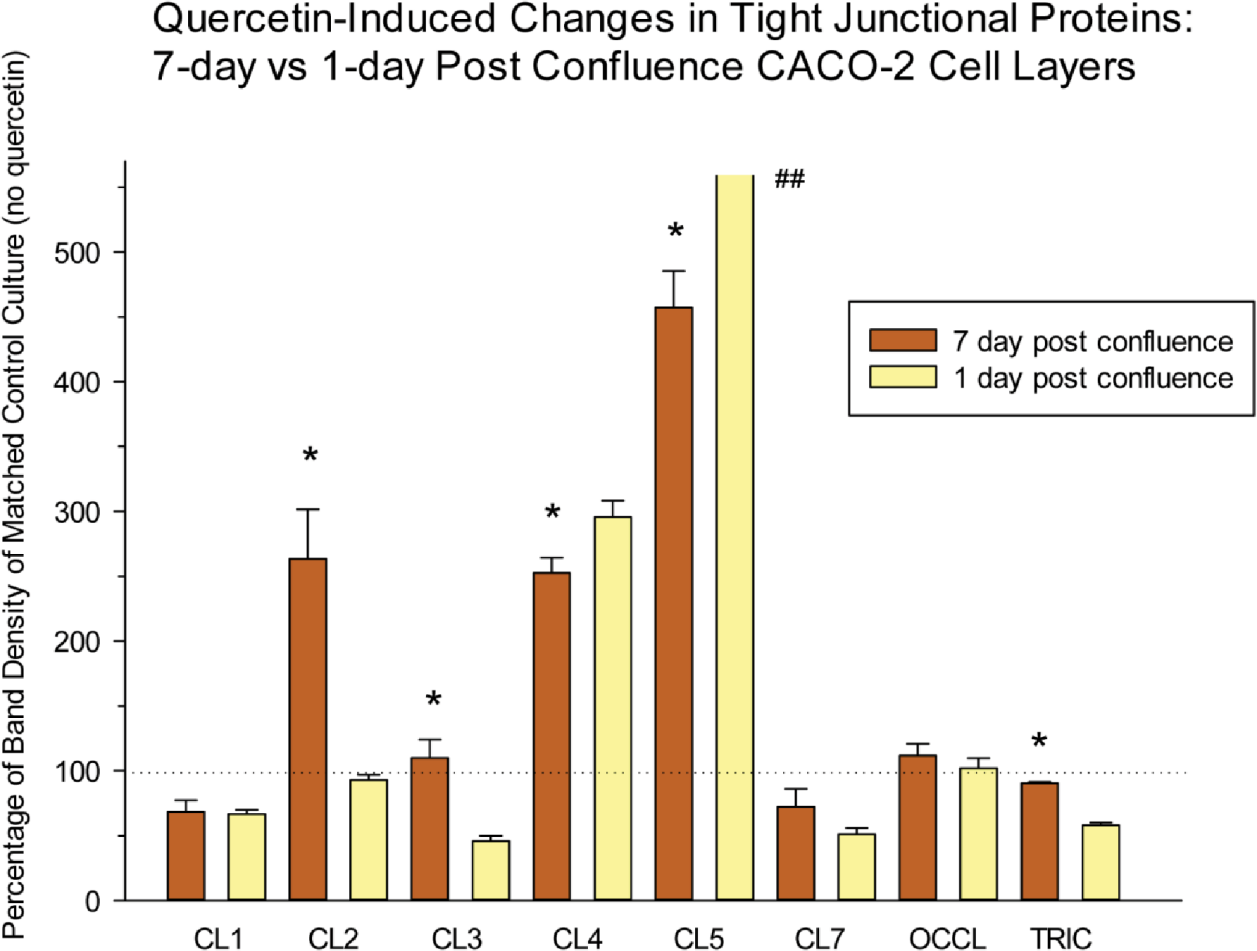
Differential effect of quercetin treatment on the tight junctional proteins of 7-day vs. 1-day post-confluent CACO-2 cell layers. 1-day and 7-day post-confluent CACO-2 cell layers in Falcon 75 cm^2^ culture flasks were refed with control medium or medium containing 400μM quercetin 48 hrs before harvesting in lysis buffer. Further steps were performed as described in Table 1. Data represents the percentage of band density of the no-quercetin control for that CACO-2 cell layer. Data shown is expressed as the mean ± standard error for an n = 3 cell layers in all cases. * indicates P < 0.05 for 1-day vs. 7-day changes in TJ protein (Student’s t test, two-tailed.

Not only are the TJ complexes changing as the differentiation process proceeds, but the response of the TJ complexes to the various micronutrients seems highly dependent on the state of differentiation at the time that the cell layers are exposed to the micronutrients. As shown in Fig 4, response of the cell layer to supplemental zinc—as evidenced by elevated R_t_—is significant for 7- and 21-day cell layers, but not 3-day cell layers, where no effect is observed. This pattern of functional change contrasts sharply with the effects of supplemental zinc on the abundance of the various integral TJ proteins (Fig 5). Zinc markedly and significantly affects the abundance of seven of the eight TJ proteins assayed in 3-day cell layers, but is virtually without effect on the TJ protein abundances of 7-day and 21-day cell layers, where effects are relatively muted. This would appear to be a general phenomenon, and not specific for any single micronutrient. Similar to zinc, quercetin’s effects on TJ protein abundance could be quite different for just-confluent vs. 7-days-post-confluent cell layers (Fig 6). Here, there were significant differences between just-confluent and 7-day-post-confluent cultures regarding claudins -2, -3, -4, -5 and tricellulin. For claudin-2, there was no effect of quercetin in just-confluent cell layers, but a dramatic 150% increase for 7-day cell layers. On the other hand, tricellulin was not affected by quercetin in 7-day cell layers, but its abundance decreased nearly 50% in just-confluent cell layers.

### The Effect of the Cell Layer Substratum on the Response to Micronutrients

As another important procedural note in performing barrier integrity studies like those described here, we observed that the membrane on which the cell layer is supported quantitatively affects the barrier integrity changes brought about by the micronutrients. Both the Millicell HA and PCF units in use in these studies provide for a cell layer sitting on a permeable substratum. However, the Millicell PCF and HA units differ markedly in the composition of the filter layer on which the epithelial layers rest. The PCF unit has a polycarbonate filter base, whereas the HA unit has a cellulose-derived filter base. Seeding cells at identical densities (1.20 x 10^5^/cm^2^) into the two different (4.2 cm^2^) units on the same day, and incubating the layers for the same (7 day) period of time, resulted in substantially different barrier properties (Table 3). Reflecting both R_t_ and J_m_, those cell layers on PCF filters could be viewed as nearly twice as “tight” as the cell layers on HA filters. Interestingly the I_sc_ values for the two different groups of cell layers were no different.

**Table 3:** Effect of the Filter Substratum on CACO-2 Transepithelial Barrier Function Properties.

	Millicell HA	Millicell PCF
**Transepithelial Resistance**	215 + 7***	363 + 16
**Short Circuit Current**	2.7 + 0.2 (NS)	2.7 + 0.1
**Transepithelial Mannitol Flux**	1.9 + 0.2***	0.9 + 0.1

CACO-2 cell layers were seeded at identical densities on Millicell PCF and Millicell HA units as described in Materials and Methods. After 7 days, cell layers were refed in apical and basal-lateral compartments with control medium prior to electrical measurements and ^14^C-D-mannitol flux studies. Data shown is expressed as the mean ± standard error of 8 cell layers per condition (2 experiments, 4 cell layers per condition).

NS indicates non significance.

*** indicates P<0.001 (Student’s t test, two-tailed).

**Fig 7:**
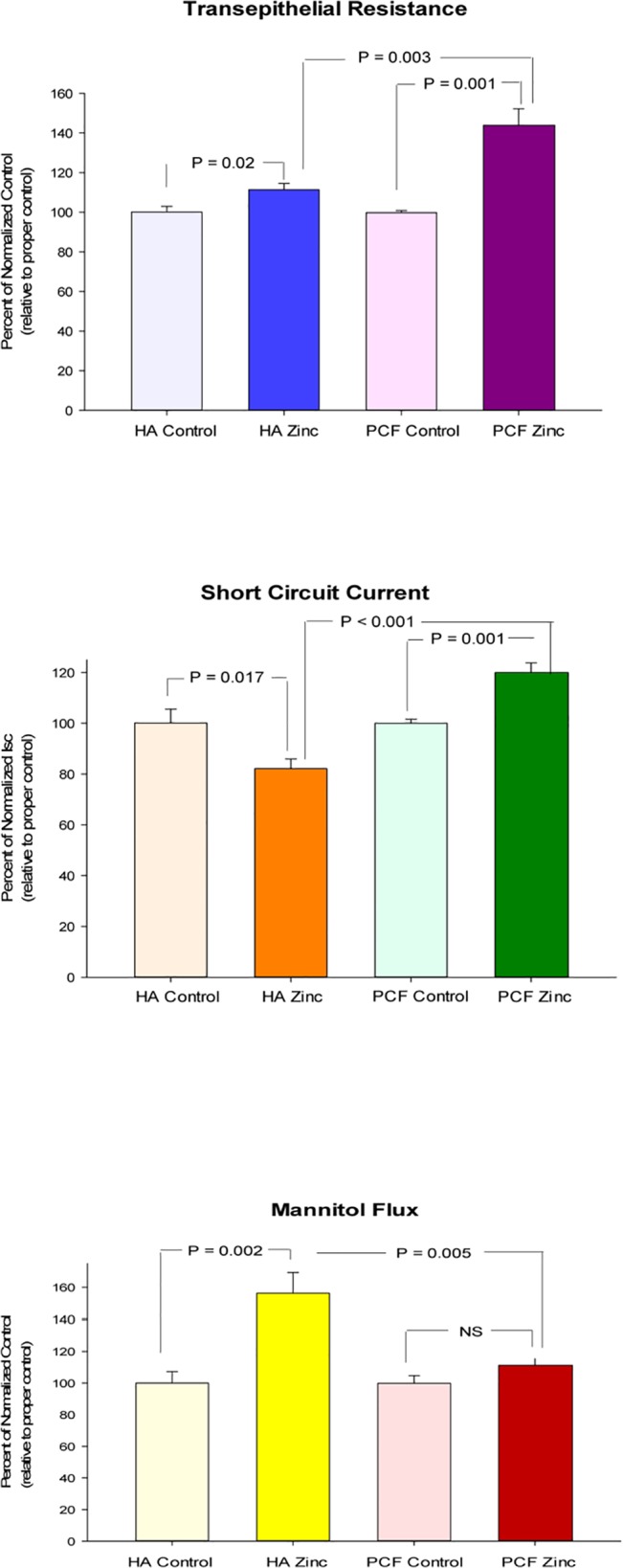
The effect of the cell layer substratum on CACO-2 epithelial barrier’s response to zinc CACO-2 cell layers were seeded at identical densities on Millicell PCF and Millicell HA units as described in Materials and Methods. After 7 days, cell layers were refed in apical and basal-lateral compartments with control medium or medium containing 100μM zinc, 48 hrs before electrical measurements. Data represents the percentage of normalized control resistance, normalized control short circuit current, and normalized control flux rate (each relative to proper control; 2 experiments, 4 cell layers per condition). Data shown is expressed as the mean ± standard error of 8 cell layers per condition. P values (Student’s t test, two-tailed) are indicated for statistical comparisons of the various conditions.

For the purposes of this study however, it was more pertinent that the response of the cell layers to supplemental zinc was quite different, depending on the filter used. Cell layers on HA filters showed a much smaller R_t_ increase in response to zinc treatment, and actually increased their permeability to mannitol, whereas those on PCF filters exhibited a much greater R_t_ increase and did not increase their permeability to mannitol (Fig 7). More pointedly, cell layers on HA filters decreased their I_sc_ by almost 20% in response to supplemental zinc, whereas cell layers on PCF filters increased their I_sc_ by over 20% in response to supplemental zinc. Along with degree of differentiation, epithelial substratum thus appears to also play a likely regulatory effect on the action of micronutrients with regard to epithelial barrier properties.

### The Effect of Butyrate on Claudin-2 and Claudin-7 Gene Expression

CACO-2 cell layers cultured for 72-hours in the presence of 5.0 mM butyrate displayed a 90% decrease in claudin-2 and a 376% increase in claudin-7 protein expression, as determined by Western immunoblot (Table 1). The claudin-2 and -7 genes were chosen as a test case to determine whether transcriptional changes necessarily corresponded with the robust changes observed on the protein level. Gene expression for claudin-2 and claudin-7 in butyrate-treated CACO-2 cell layers was assessed by RT-PCR at 4, 12, 24, 48, and 72 hours after treatment. Significant changes were not observed in claudin-2 or claudin-7 gene expression in butyrate- treated cell layers compared to time-matched control cell layers (data not shown). These results demonstrated that butyrate-mediated regulation of TJ protein abundance is likely not occuring at the transcriptional level.

## Discussion

In our previously published work we reported on the effect of various micronutrients on LLC-PK_1_ renal epithelial cell layers and TJs [8]. As in our current study on human gastrointestinal CACO-2 epithelial cell layers, butyrate, indole, zinc and quercetin all significantly increased R_t_ across LLC-PK_1_ cell layers, although zinc and especially indole had greater effects on CACO-2 R_t_ than was observed for LLC-PK_1_. Quercetin was able to significantly reduce mannitol diffusion across LLC-PK_1_ cell layers, but not CACO-2 cell layers. However, it is the overall pattern of micronutrient-induced changes in TJ protein abundance where the differences between effects on CACO-2 cell layers vs. LLC-PK_1_ cell layers are most striking (see Table 1 of the current study vs. Table 2 of Mercado et al [2013])[8]. As just two examples, quercetin induces a 164% increase in claudin-2 levels in CACO-2 cell layers, but *decreased* claudin-2 levels of LLC-PK_1_ cell layers by 76%. Butyrate dramatically increased claudin-7 levels of CACO-2 cell layers by 376%, yet decreased claudin-7 levels of LLC-PK_1_ cell layers by 36%. On the other hand, zinc induces no change in 7-day CACO-2 cell layer claudin-5 levels and yet increased claudin-5 levels of LLC-PK_1_ cell layers by a striking 136%. The more noteworthy similarities and differences between micronutrient effects on CACO-2 vs LLC-PK_1_ cell layers are summarized concisely in Tables 4 and 5 for transepithelial physiology and TJ proteins, respectively. In summary, the effects of any single micronutrient on TJ proteins appear to be highly dependent on the specific epithelial cell layer under study.

**Table 4:** Points of Similarity and Dissimilarity in the Effects of Micronutrients on Barrier Function in the LLC-PK1 Renal Cell Layer vs the CACO-2 Gastrointestinal Cell Layer.

Transepithelial Resistance	LLC-PK_1_	CACO-2
100 uM Zinc	34% increase*	60% increase*
400 uM Quercetin	42% increase*	No Change
1 mM Indole	24% increase*	50% increase*
Mannitol Diffusion		
100 uM Zinc	No Chnage	60% increase*
400 uM Quercetin	21% decrease*	No Change
1 mM Indole	No Change	No Change

* Indicates a statistically significant change

**Table 5:** Points of Similarity and Dissimilarity in Micronutrient Effects on Claudin Levels in the LLC-PK1 Renal Cell Layer vs the CACO-2 Gastrointestinal Cell Layer.

Similarity	LLC-PK_1_	CACO-2
100 uM Quertin on CL-5	186% increase*	357% increase*
1 mM Indole on CL-5	163% increase*	62% increase*
1 mM Indole on CL-2	50% decrease*	28% decrease*
Disimilarity		
400 uM Quercetin on CL-7	209% increase*	No Change
100 uM Zinc on CL-5	136% increase*	No Change
100 uM Zinc on CL-7	58% increase*	No Change
400 uM Quercetin on CL-2	76% decrease*	164% increase*
100 uM Zinc on CL-4	36% decrease*	No Change

* Indicates a statistically significant change

The mechanism for micronutrient-induced change in epithelial barrier function and alteration of TJ protein levels may not necessarily involve changes in select TJ gene transcription. In our study, differentiated CACO-2 cell layers displayed dramatic changes in the level of claudin-2 (90% decrease) and claudin-7 (376% increase) protein as a result of butyrate treatment. However, this modulation of claudin-2 and claudin-7 protein abundance was seemingly not the result of altered levels of gene transcription. Altered regulation of these two proteins in response to butyrate apparently occurred at the post-transcriptional level. In this regard it is worth noting that although butyrate is a “differentiating agent” in the CACO-2 model, its effects can be somewhat different than cellular changes achieved through spontaneous differentiation [31]. Ma et al (2012)[32] have recorded enhanced occludin expression in IPEC-J2 intestinal epithelia, and Wang et al. (2012)[17] observed enhanced claudin-1 transcription in cdx2-IEC intestinal epithelial cells also as a result of butyrate treatment. However, improvement of barrier function in CACO-2 cell layers has also been seen in the absence of changes in TJ gene transcription [16]. Here butyrate-induced changes in posttranslational regulation, such as phosphorylation and translocation, have been invoked. It is worth noting that butyrate-induced changes in CACO-2 proteasomal activity have been observed[33,34], as have butyrate- induced changes in CACO-2 lipoxygenase activity linked to TJ permeability [35]. Lipoxygenase regulation of proteasomal activity has been observed (Whitehouse and Tisdale, 2001). We are very aware that we report transcriptional data only for one micronutrient, butyrate, and for only two TJ gene transcripts, claudin-2 and -7. The situation may be quite different for other micronutrients and for other TJ genes and proteins.

As stated in the Introduction, our present study builds on earlier reports that documented each of the six micronutrients under study to have barrier-enhancing effects, often in the CACO-2 cell model under study here. Butyrate enhancement of barrier integrity has recently been reviewed [36]. Butyrate, and other short chain fatty acids, were shown to not only enhance TJ barriers basally, but also to alleviate the TJ damage brought about by pathology as diverse as that induced by exposure of CACO-2 cell layers to ethanol and *Campylobacter jejuni* [35,37,38]. These effects of butyrate may be mediated by protein kinase A and may involve stimulation of differentiation [16]. Indole, another bacterial metabolite, was earlier reported to modify TJs and improve barrier integrity in the human GI cell line, HCT-8. This may arise from indole effects on endogenous cytokine synthesis, secretion and action [19]. Similar results were shown for mouse intestine [39]. Quercetin, the most widely prevalent flavonoid in the diet, increased R_t_ across CACO-2 cell layers, and this coincided with an increase in claudin-4 levels [26]. This action of quercetin may be mediated through protein kinase C-delta [25]. Quercetin has demonstrated protective action against the barrier-disruptive effects of TNF-alpha, indomethacin and hydrogen peroxide [27,40,41]. The plant alkaloid, berberine, was also earlier reported to improve barrier properties of CACO-2 cell layers [24]. Berberine also protects against TNF-alpha-induced barrier compromise of HT-29/B6 epithelial layers as well as rat colon mucosa, effects linked to tyrosine kinase inhibition [23,42,43]. Berberine’s effects on reducing myosin light chain kinase expression and consequently myosin light chain phosphorylation, may also be involved in its barrier enhancing activity [42]. Zinc may actually have some of the oldest published literature showing barrier protection by a micronutrient. Sturniolo et al. (2002)[14] showed that rats with chemically-induced colitis exhibited a lower number of small intestinal TJs that were permeable to lanthanum when the rats were supplemented orally with zinc. Oral zinc supplementation likewise modified intestinal TJs and augmented barrier integrity in pigs [12]. Our own group had earlier shown zinc-induced TJ modification and R_t_ elevation in CACO-2 cell layers [11]. It is worth noting however that the well known anti-diarrheal actions of zinc are not simply due to reduction of intestinal paracellular leak, but also arise from zinc inhibition of enterocyte basal-lateral K^+^ channels [44]. It is noteworthy too that although the effects of micromolar zinc levels on TJs appear to require sustained exposure, millimolar concentrations of zinc have been observed to exert very rapid reduction of paracellular conductance [11,45]. We observed efficacy in TJ barrier enhancement by all of our selected micronutrients with the exception of nicotine. Nicotine had been previously reported to modify TJ proteins and reduce paracellular permeability of CACO-2 cell layers, reflecting the action of cigarette smoking in reducing GI permeability [22]. We speculate that differences among substrains of the CACO-2 cell line in use by different laboratories (as arise spontaneously during continued weekly passaging of any cell line), may explain our inability to have observed nicotine effects on R_t_ or J_m_ at similar concentrations in our present study.

## Conclusion

There are four central conclusions from this current study:

1. Although there was an overall similarity in improving epithelial barrier integrity generally, each of the 5 micronutrients exhibiting positive effects on barriers appeared to perform this function uniquely, with signature-like effects on the cells’ TJ protein complement.
2. The actions of each effective micronutrient are moreover likely to be highly tissue-specific, as seen by the very different effects of zinc, quercetin, indole and butyrate on the renal (LLC-PK_1_) epithelial cell layer model vs. the gastrointestinal (CACO-2) epithelial cell layer model.
3. The fact that five chemically distinct compounds induce their own, unique, signature-like changes in TJ protein abundance, while all induce decreased TJ permeability to one or more solute classes, underscores the regulatory complexity of the TJ complex, and the likely high number of permeability “states” that a given TJ complex can exist in. For example, quercetin up-regulated claudin-2 levels by over 160% in CACO-2 cell layers and yet also increased R_t_, a true cautionary note to those who think that claudin-2 is simply synonymous with ion conductance.
4. The exact effects of any given micronutrient on the barrier properties of the epithelial cell layer and on its complement of TJ proteins, are likely to be dependent upon the state of differentiation of the epithelial cell layer, as well as the composition of its substratum, at the time of exposure to the supplemental micronutrient.

These conclusions have, in turn, two broad and related biomedical implications: a) by achieving additive effects on TJ complexes, combinations of micronutrients, may (but by no means necessarily) achieve superior therapeutic results (relative to those induced by individual micronutrients) in GI diseases that are characterized in part by barrier dysfunction; and b) considering the range of enterocyte differentiation states that would exist in a section of gastrointestinal mucosa partially damaged in IBD, a combination of micronutrients may be able to extend beneficial barrier effects across this variable range of differentiation states, thereby providing maximal barrier benefit to the tissue at large.

As we outlined in our previous study, there are intrinsic clinical dividends to research on altering TJ composition and permeability by simple and commonplace dietary compounds, as opposed to more targeted approaches utilizing, for example, oligonucleotides or small inhibitory RNAs to achieve “designer” TJ complexes. Given that the TJ complex consists of approximately 30 (known) barrier TJ proteins (in addition to all of the intracellular TJ-associated proteins), and that these proteins function in zipper-like fashion through a variety of homotypic and heterotypic interactions [46], one has a potential set of over 400 possible interactional pairings, a significant percentage of which are likely to change in any attempt at altering TJ composition by rational design. If only *10* unique TJ proteins were to exist in a particular cell layer, one is still confronted with 45 possible interactional pairings, a situation that may allow for many unexpected and unwanted permeability effects in addition to one’s desired effect. If one seeks to obtain a “better TJ complex” by rational design directed at simply up-regulation or down-regulation of a particular individual TJ protein (tabling for the moment the reality that these individual proteins can be modified by their phosphorylation state as well), one is confronted by an unwieldy set of possibilities. This complexity is one reason why we utilize naturally-occurring TJ modulators vetted by evolution, i.e. the micronutrients. Human cells and tissues have been in contact with substances such as zinc, indole, butyrate and quercetin for millennia. If these substances have a positive effect on epithelial barriers in our tissues, it is an adaptational process that has been worked out over eons. Moreover, if the actions of one of these substances are salutary for a targeted epithelial layer’s barrier integrity, this action very likely evolved without detriment to other epithelial organ systems by the very nature of it being an adaptational advantage to the entire organism. Given that we have shown that inducers of TJ modification are highly cell- and tissue-specific, this factor could be crucial in eventual clinical application. The same recommendation would not be conferred with a novel molecular biological approach or classical synthesized drug that would be discovered to have gastrointestinal barrier-enhancing effects at the lab bench.

## Supporting Information

S1 Fig Complete Claudin-2 Western Immunoblot of 3-day, 7-day and 21-day CACO-2 Cell Layers. (PDF)

S2 Fig Effect of Berberine on Claudin-1 in CACO-2 Cell Layers. (PDF)

S3 Fig Effect of Berberine on Claudin-2 in CACO-2 Cell Layers. (PDF)

S4 Fig Effect of Berberine on Claudin-3 in CACO-2 Cell Layers. (PDF)

S5 Fig Effect of Berberine on Claudin-4 in CACO-2 Cell Layers. (PDF)

S6 Fig Effect of Berberine on Claudin-5 in CACO-2 Cell Layers. (PDF)

S7 Fig Effect of Berberine on Claudin-7 in CACO-2 Cell Layers. (PDF)

S8 Fig Effect of Berberine on Occludin in CACO-2 Cell Layers. (PDF)

S9 Fig Effect of Berberine on Tricellulin in CACO-2 Cell Layers. (PDF)

S10 Fig Effect of Quercetin on Claudin-5 in 7-Day Old CACO-2 Cell Layers. (PDF)

S11 Fig Effect of Quercetin on Claudin-5 in 1-Day Old CACO-2 Cell Layers. (PDF)

S12 Fig Effect of Quercetin on Occludin in 7-Day Old CACO-2 Cell Layers. (PDF)

S13 Fig Effect of Quercetin on Occludin in 1-Day Old CACO-2 Cell Layers. (PDF)
