# Supplementary figures and images for: Remodeling of Tight Junctions and Enhancement of Barrier Integrity of the CACO-2 Intestinal Epithelial Cell Layer by Micronutrients
Source: PLoS One. 2015 Jul 30;10(7):e0133926. doi: 10.1371/journal.pone.0133926 (PMC4520484; doi:10.1371/journal.pone.0133926)

A

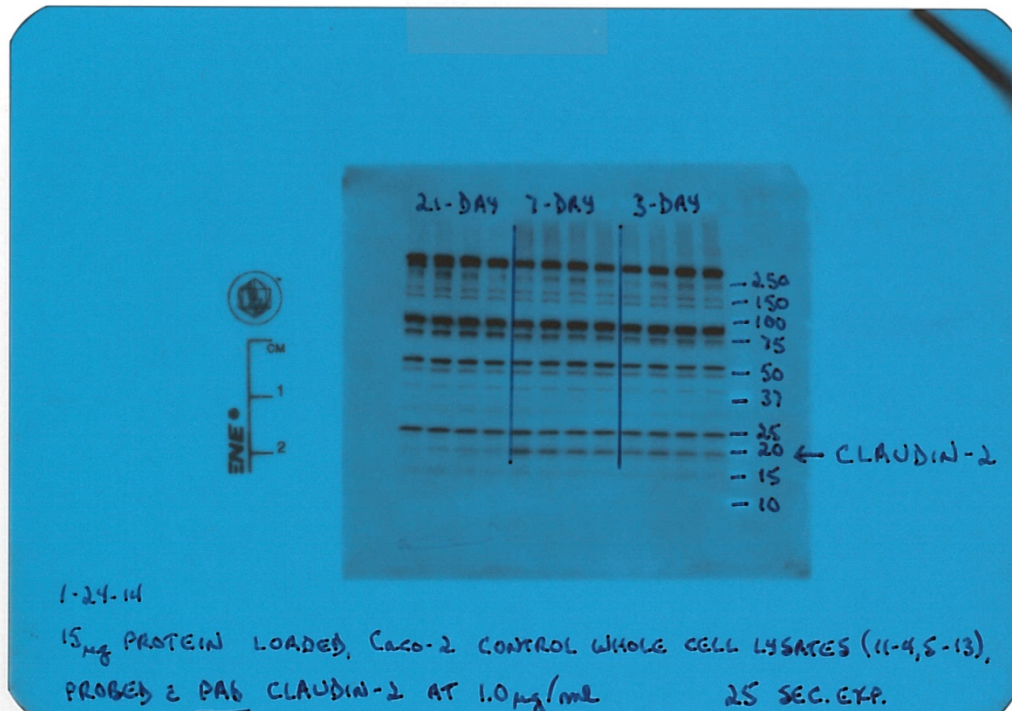

Complete Claudin-2 Western Immunoblot of 3-day, 7-day and 21-day CACO-2 Cell Layers

Supplement: S1 Fig — (PDF) [file pone.0133926.s001.pdf]

B

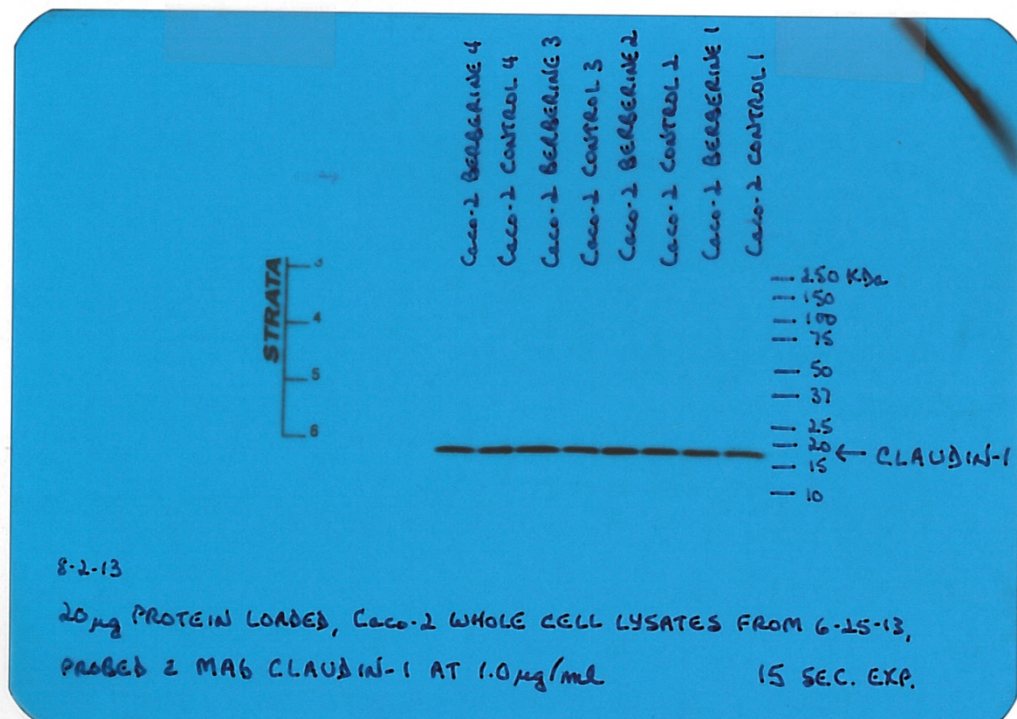

Effect of Berberine on Claudin-1 in CACO-2 Cell Layers

Supplement: S2 Fig — (PDF) [file pone.0133926.s002.pdf]

C

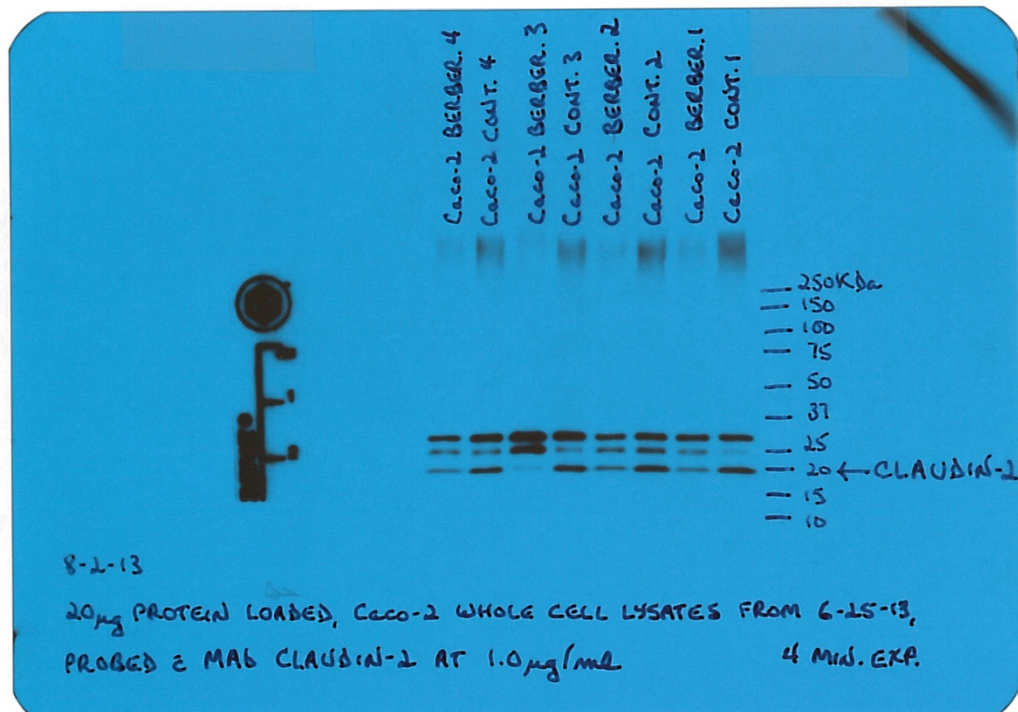

Effect of Berberine on Claudin-2 in CACO-2 Cell Layers

Supplement: S3 Fig — (PDF) [file pone.0133926.s003.pdf]

D

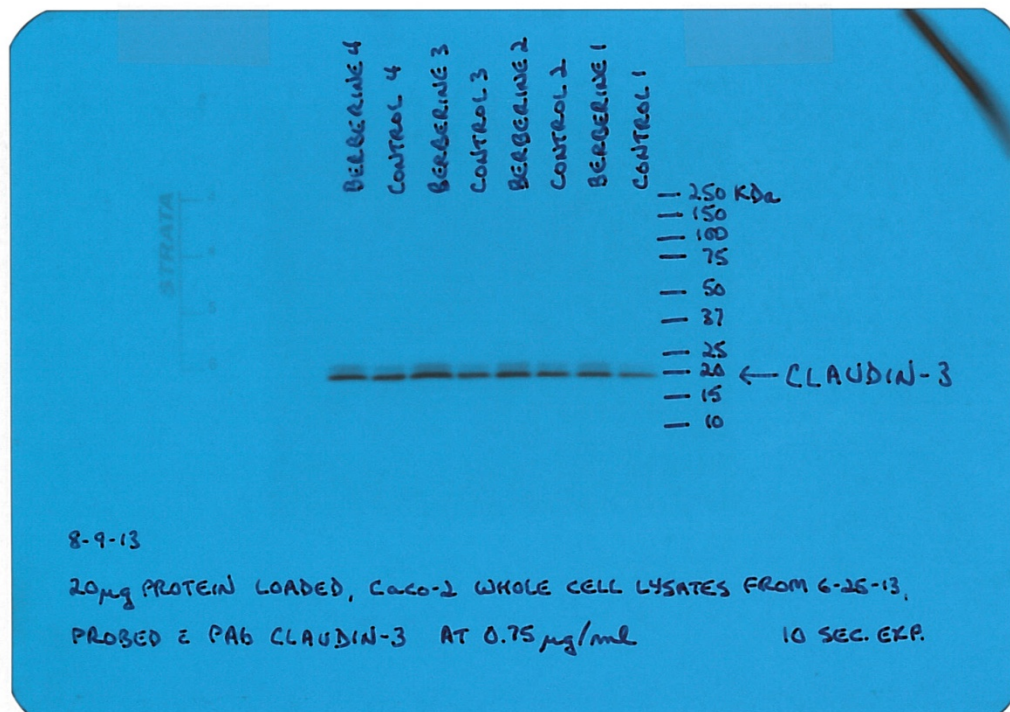

Effect of Berberine on Claudin-3 in CACO-2 Cell Layers

Supplement: S4 Fig — (PDF) [file pone.0133926.s004.pdf]

E

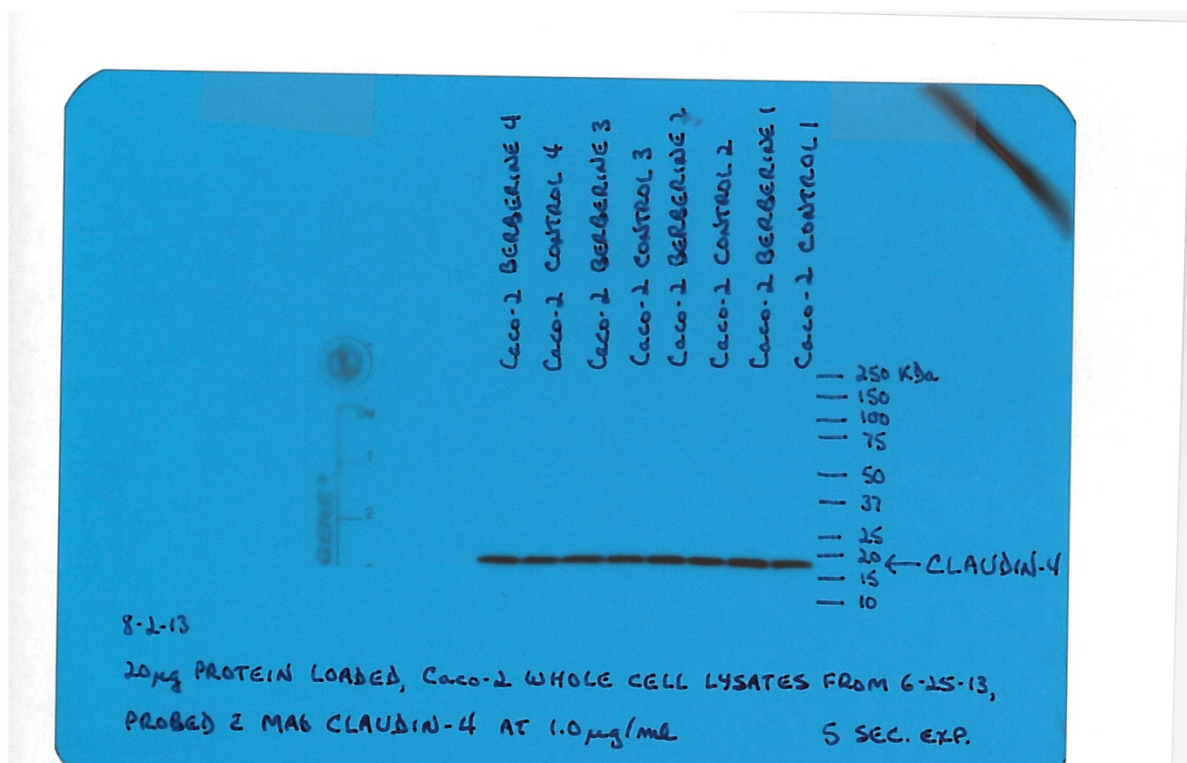

Effect of Berberine on Claudin-4 in CACO-2 Cell Layers

Supplement: S5 Fig — (PDF) [file pone.0133926.s005.pdf]

F

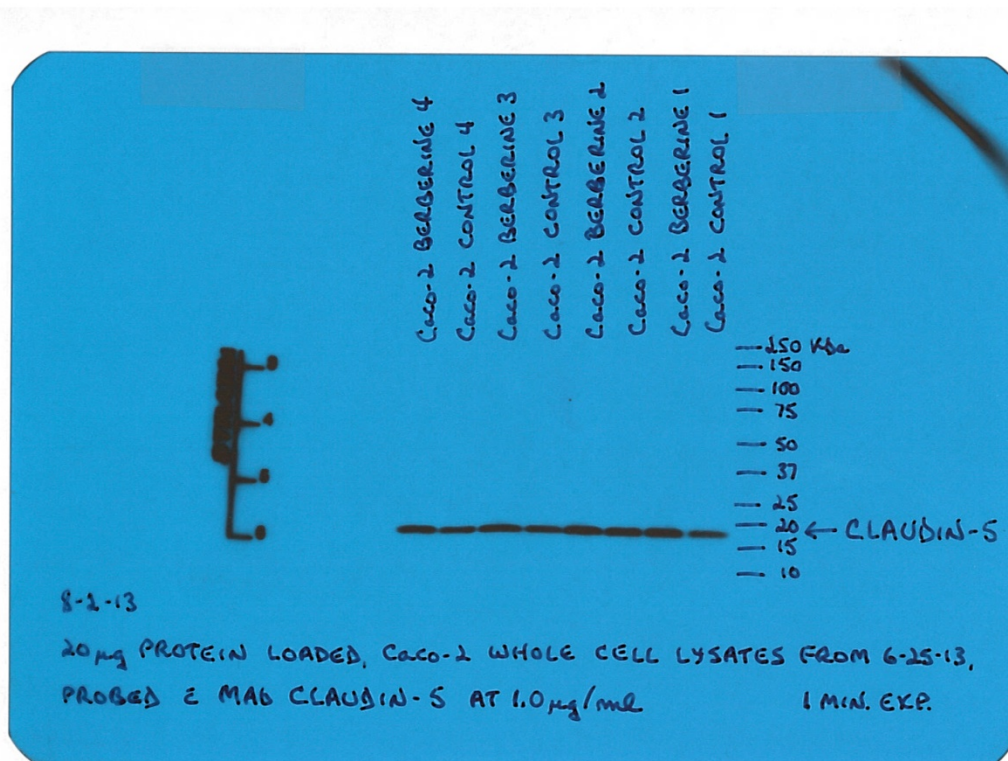

Effect of Berberine on Claudin-5 in CACO-2 Cell Layers

Supplement: S6 Fig — (PDF) [file pone.0133926.s006.pdf]

G

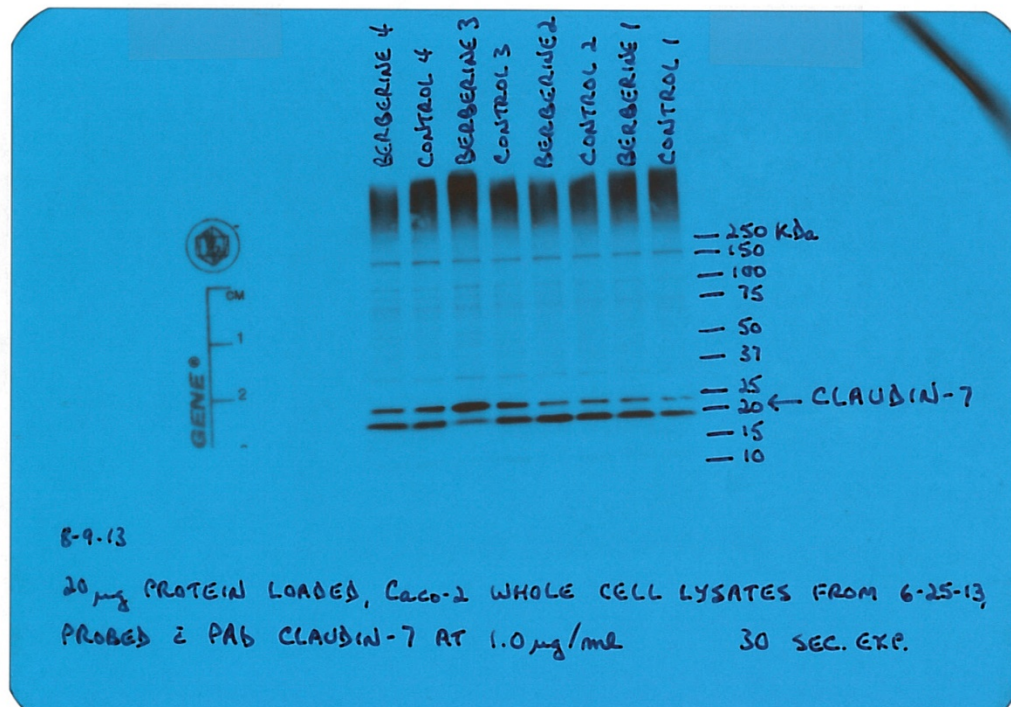

Effect of Berberine on Claudin-7 in CACO-2 Cell Layers

Supplement: S7 Fig — (PDF) [file pone.0133926.s007.pdf]

H

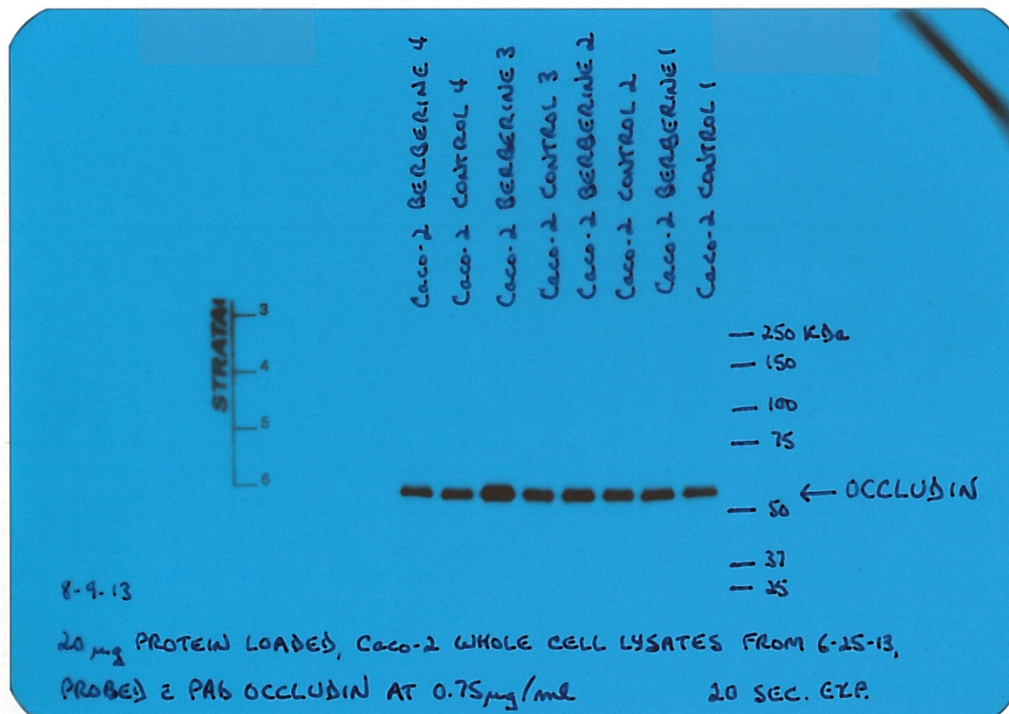

Effect of Berberine on Occludin in CACO-2 Cell Layers

Supplement: S8 Fig — (PDF) [file pone.0133926.s008.pdf]

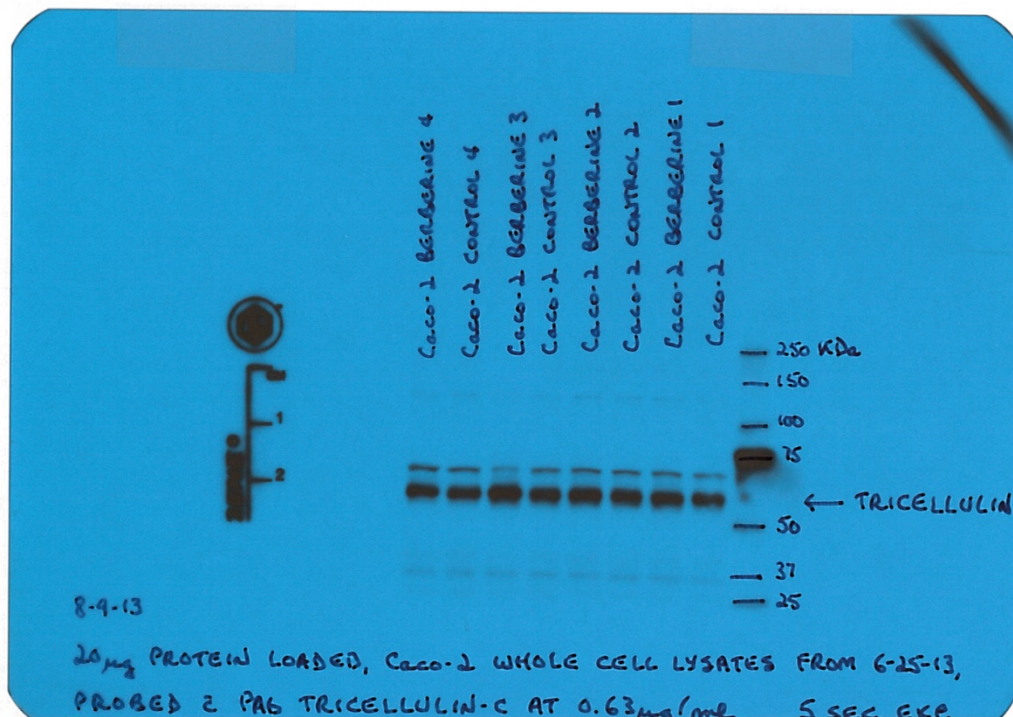

Effect of Berberine on Tricellulin in CACO-2 Cell Layers

Supplement: S9 Fig — (PDF) [file pone.0133926.s009.pdf]

J

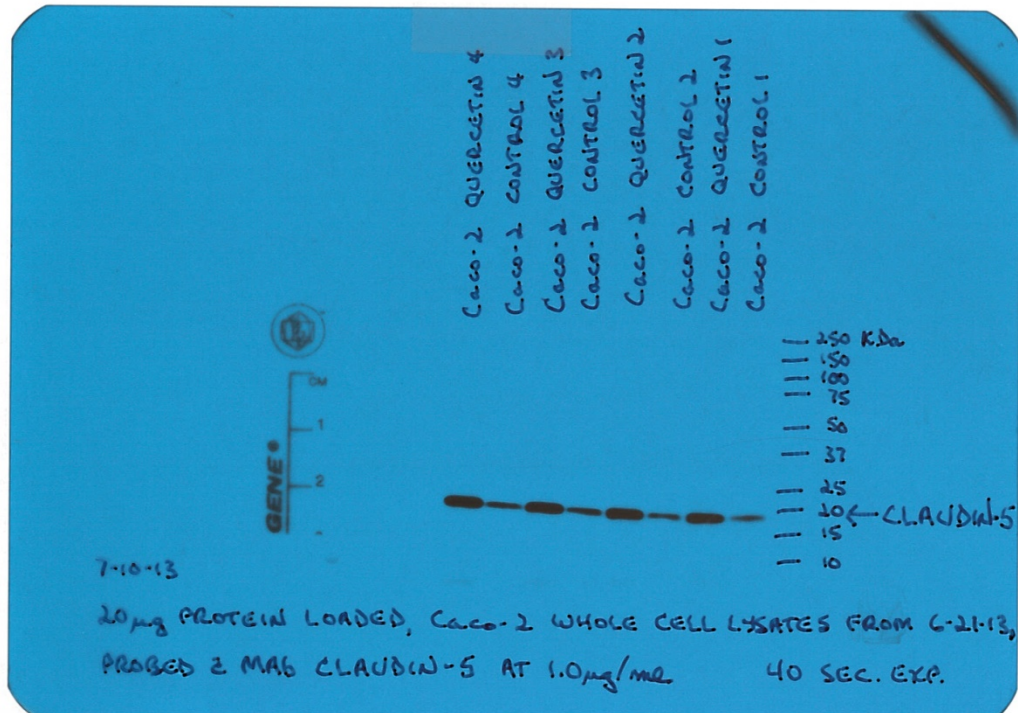

Effect of Quercetin on Claudin-5 in 7-Day Old CACO-2 Cell Layers

Supplement: S10 Fig — (PDF) [file pone.0133926.s010.pdf]

K

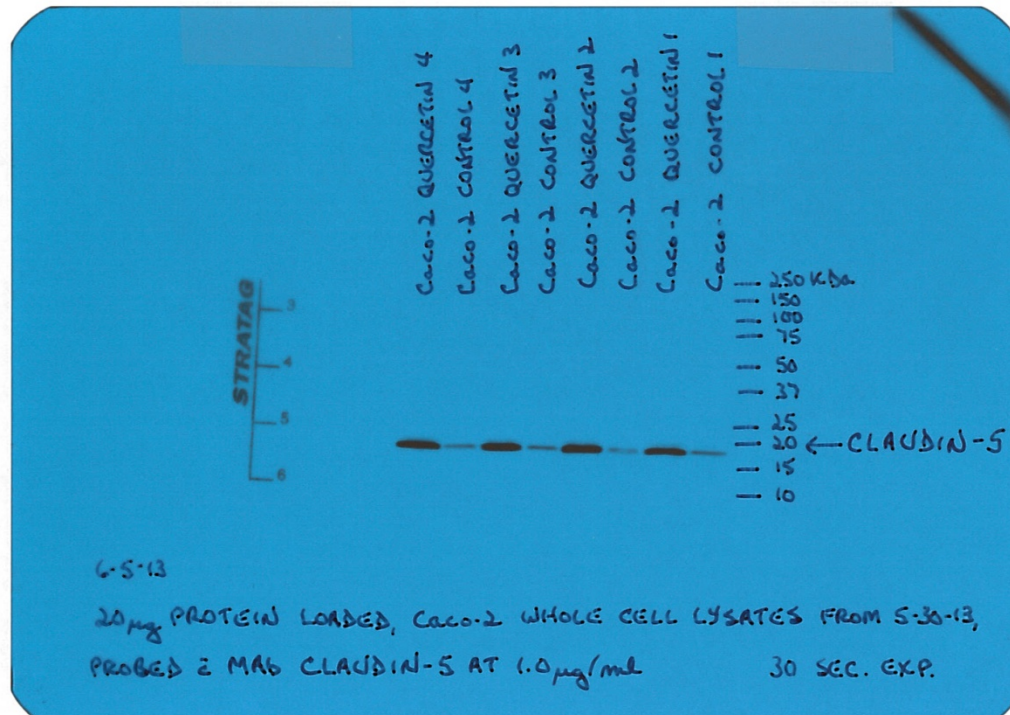

Effect of Quercetin on Claudin-5 in 1-Day Old CACO-2 Cell Layers

Supplement: S11 Fig — (PDF) [file pone.0133926.s011.pdf]

L

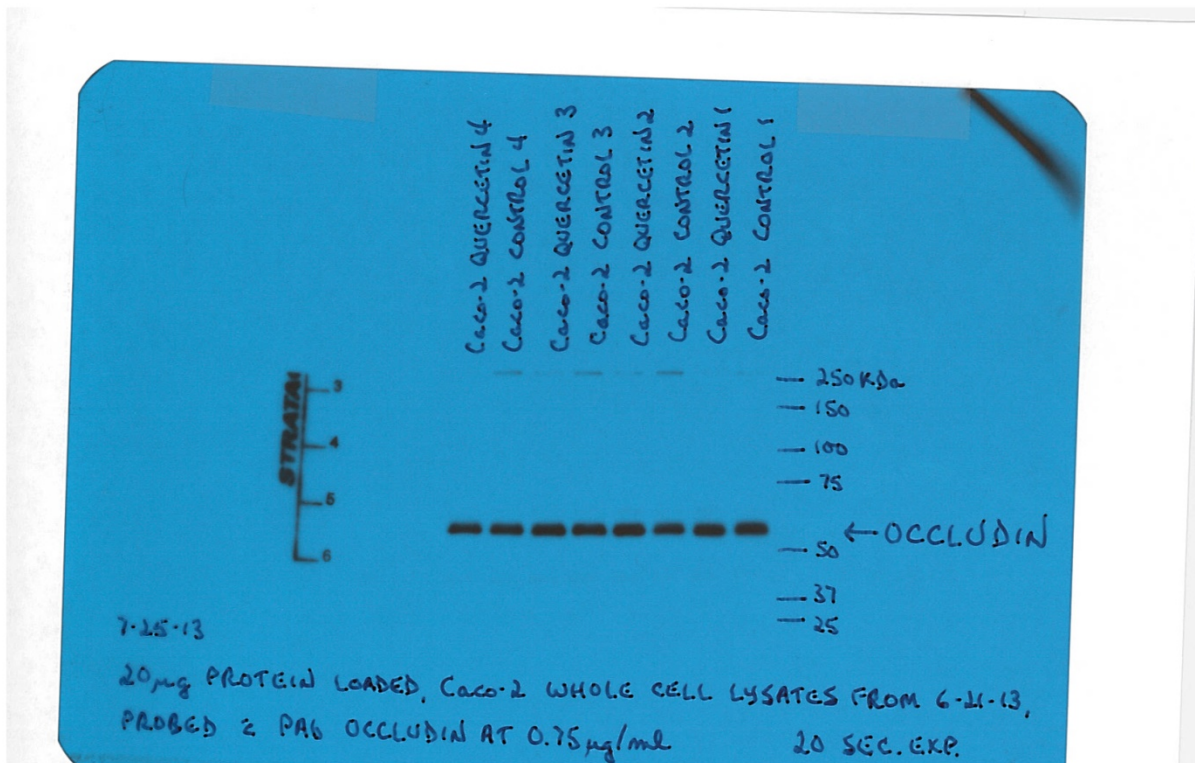

Effect of Quercetin on Occludin in 7-Day Old CACO-2 Cell Layers

Supplement: S12 Fig — (PDF) [file pone.0133926.s012.pdf]

M

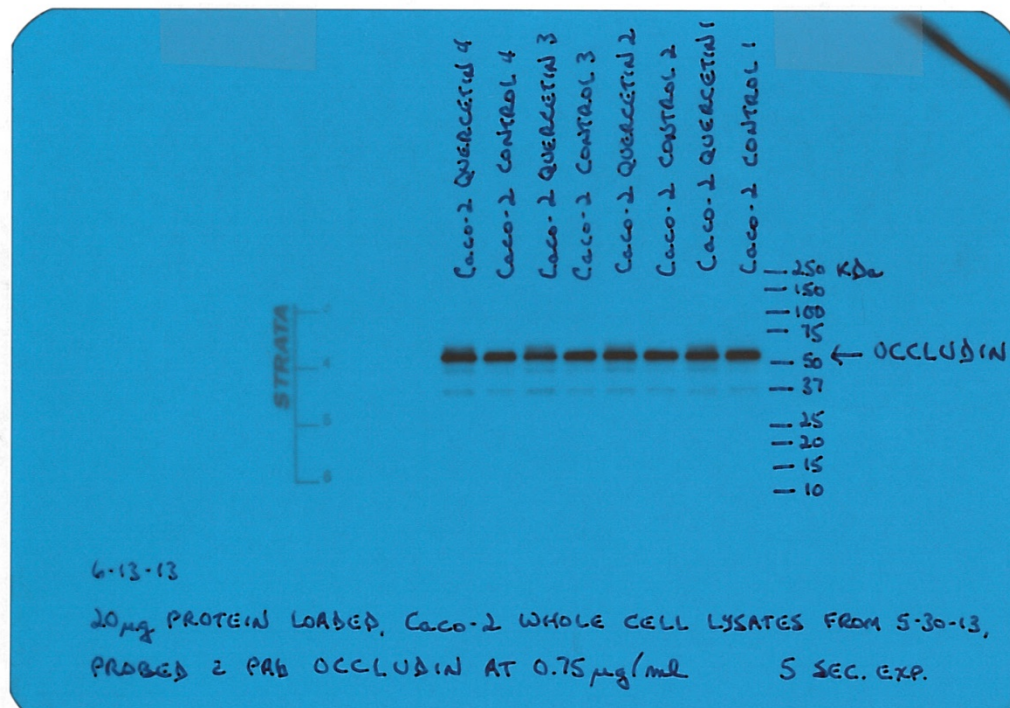

Effect of Quercetin on Occludin in 1-Day Old CACO-2 Cell Layers

Supplement: S13 Fig — (PDF) [file pone.0133926.s013.pdf]
